# Radiographically confirmed community-acquired pneumonia in hospitalized adults due to pneumococcal vaccine serotypes in Sweden, 2016–2018—The ECAPS study

**DOI:** 10.3389/fpubh.2023.1086648

**Published:** 2023-02-17

**Authors:** Karin Hansen, Elisabeth Rünow, Gustav Torisson, Christian Theilacker, Andreas Palmborg, Kaijie Pan, Qin Jiang, Jo Southern, Rohini Beavon, Bradford D. Gessner, Kristian Riesbeck, Jonas Ahl

**Affiliations:** ^1^Section of Infectious Diseases, Department Translational Medicine, Faculty of Medicine, Lund University, Malmö, Sweden; ^2^Section of Clinical Microbiology, Department Translational Medicine, Faculty of Medicine, Lund University, Malmö, Sweden; ^3^Vaccines Global Medical Development, Scientific and Clinical Affairs, Pfizer, Collegeville, PA, United States; ^4^Pfizer Vaccines, Medical Development, Scientific and Clinical Affairs, Stockholm, Sweden

**Keywords:** community-acquired pneumonia (CAP), pneumococcal conjugate vaccine, pneumococcal vaccination, *Streptococcus pneumoniae*, Sweden

## Abstract

**Objectives:**

In Sweden, pneumococcal serotype distribution in adults with community-acquired pneumonia (CAP) and potential coverage of currently licensed pneumococcal conjugate vaccines (PCVs) is unknown.

**Methods:**

During 2016–2018, patients aged ≥18 years hospitalized with radiologically confirmed (RAD+) CAP were enrolled at Skåne University Hospital in a study on the etiology of CAP in Sweden (ECAPS). Urine samples and blood cultures were collected per-protocol. *Streptococcus pneumoniae* (Spn) culture isolates were serotyped and urine samples tested for the pan-pneumococcal urinary antigen (PUAT) and multiplex urine antigen detection (UAD) assay, detecting 24 serotypes.

**Results:**

Analyses included 518 participants with RAD+CAP; 67.4% were ≥65 years of age, 73.4% were either immunocompromised or had an underlying chronic medical condition. The proportion of CAP due to Spn identified by any method was 24.3% of which 9.3% was detected by UAD alone. The most frequently identified serotypes were 3 (26 cases, 5.0% of all CAP), and 8, 11A and 19A (10 cases each, 1.9%). In individuals aged 18–64 and ≥65 years, respectively, PCV20 serotypes contributed to 35 of 169 (20.7%) and 53 of 349 cases of all CAP (15.2%), and PCV13 serotypes caused 21 of 169 (12.4%) and 35 of 349 (10.0%) cases. PCV15 coverage was 23 of 169 (13.6%) and 42 of 349 (12.0%) in individuals aged 18–64 and ≥65 years, respectively. Overall, PCV20 increases the coverage of all CAP from 10.8% (PCV13) to 17.0%.

**Conclusion:**

Compared to earlier pneumococcal vaccines, PCV20 expands the coverage of all-cause CAP. Routine diagnostic tests underestimate the proportion of CAP caused by Spn.

## Introduction

Community-acquired pneumonia (CAP) continues to be a major cause of morbidity and mortality in adults worldwide (1). CAP incidence increases exponentially in older adults, with rates of 680 per 100,000 persons per year in adults aged 65–74 year, 1,640 per 100,000 persons per year in those aged 75–84 years and 3,460 per 100,000 per year in those aged ≥85 years (2). In addition, chronic underlying comorbidities and immunocompromising conditions predispose to CAP (3). Multiple pathogens cause CAP, but *Streptococcus pneumoniae* (Spn) remains the most frequently identified bacterial cause in adults (4). Until recently, two vaccines were licensed for protection against pneumococcal infections in adults: the 23-valent pneumococcal polysaccharide vaccine (PPV23) and the 13-valent pneumococcal conjugate vaccine (PCV13). In addition, two novel PCVs have now been licensed for adult use in the European Union: a 15-valent and a 20-valent PCV. PCV15 contains PCV13 serotypes plus serotypes 22F and 33F (5) while PCV20 contains PCV15 serotypes plus serotypes 8, 10A, 11A, 12F, and 15B (6, 7). Single dose vaccination with PCV20 or sequential vaccination of PCV15 followed by PPV23 have recently also been recommended by the American Committee for Immunization Practices (ACIP) for use in adults aged ≥65 years and in adults aged 18–64 years with underlying chronic medical conditions (CMC) (8). In Sweden, pneumococcal vaccination is recommended for adults at risk for pneumococcal disease including adults aged 65 years or older since the 1990s, but vaccine uptake has been low (3).

The burden of disease addressed by pneumococcal vaccines is a key variable for public health evaluations of these next-generation PCVs. However, studies that rely on standard of care diagnostics underestimate pneumococcal CAP burden due to low implementation of diagnostic tests and poor sensitivity of culture-based tests, especially when patients have received prior antibiotics (9). The use of a pan-pneumococcal urinary antigen test (PUAT) and a multiplex urine antigen detection (UAD) assay have been shown to increase the diagnostic detection rates for Spn (9, 10). The goal of the Etiology of CAP in Sweden (ECAPS) study was to document the proportion of radiographically confirmed CAP in adults caused by Spn vaccine associated serotypes.

## Patients and methods

### Study design, setting, and participants

This prospective population-based single-site, cohort study enrolled adults aged ≥18 years hospitalized with radiologically confirmed CAP admitted to Skåne University Hospital (SUS) in Malmö, Sweden between September 2016 and September 2018. SUS is a 600-bed tertiary referral hospital, serving a population of 400,000 inhabitants in the Malmö area and treats 64,000 patients annually. All patients presenting to the emergency department (ED) with chest imaging ordered were evaluated for eligibility. Adults aged ≥18 years living in the study catchment area with clinically suspected CAP based on the presence of at least two of 10 predefined signs or symptoms in combination with radiologic findings consistent with pneumonia as confirmed by a certified radiologist, and who were able and willing to provide a urine sample, were included in the study. Exclusion criteria were hospitalization <30 days prior to admission and previous enrolment to the study. Further information on inclusion and exclusion criteria can be found in Supplementary material. Enrolment was performed within 48 h of admission and written informed consent was obtained before any procedures were initiated.

### Microbiological testing

Blood culture, respiratory tract specimens and pleural fluid were cultured at the Clinical Microbiology (Laboratory Medicine Skåne) according to standard methods (Supplementary material). Serotypes of pneumococcal isolates was performed in the Riesbeck Laboratory using a polymerase chain reaction (PCR) and the Quellung reaction (Supplementary material). Urine specimens were tested by BinaxNOW *S. pneumoniae*^®^ (Abbott Diagnostics, Scarborough, ME), UAD1, and UAD2 at Pfizer's Vaccines Research and Development Laboratory (Pearl River, NY, for details see Supplementary material) (11, 12). The UAD1 assay is designed to capture 13 Spn serotype-specific polysaccharides excreted in human urine; 1, 3, 5, 6A, 6B, 7F, 9V, 14, 18C, 19A, 19F 23F and the UAD2 test detects an additional 11 Spn serotypes (2, 8, 9N, 10A, 11A, 12F, 15B/C, 17F, 20, 22F and 33F).

### Study procedures and assessments

Information was collected on demographic variables, medical history, clinical presentation, admission chest imaging results, pneumonia severity, hospital utilization, and mortality in-hospital, and at 30 days and 90 days post-enrolment. Classification and assessments were as outlined in Supplementary material.

### Data analysis

Patients enrolled in the study who had a final diagnosis of radiologically confirmed CAP as confirmed by a study physician (K.H., E.R.) were included in the analysis. Patients who had received any pneumococcal vaccine within 30 days of enrolment were excluded because of the potential for false positive test UAD1/UAD2 results. Patients with pneumococci cultured from the blood, lower respiratory secretions, or pleural fluid or a positive antigen test (PUAT or UAD assay) were classified as positive for Spn. Further information can be found in Supplementary material. Analysis results are based on descriptive statistics summary.

## Results

### Study population

**Figure 1:**
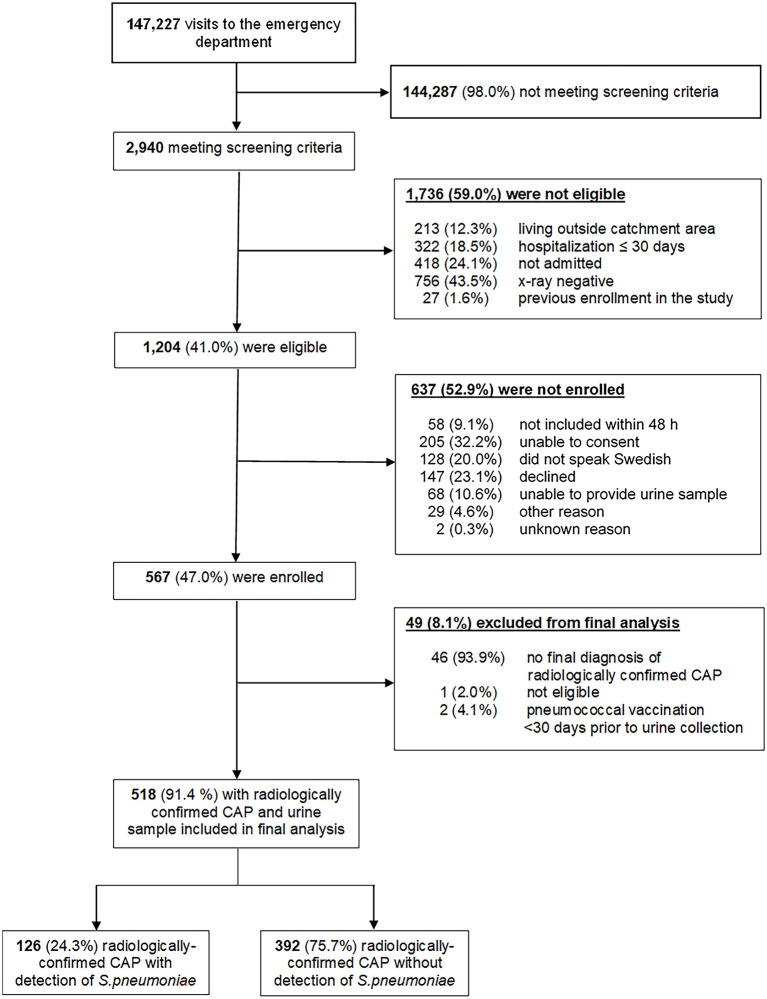
Screening, eligibility, and enrolment of patients with CAP.

All patients seeking care at the ED between September 16th, 2016 and September 16th, 2018 were screened for clinical suspicion of CAP and 1,204 patients met eligibility criteria. 567 participants were enrolled (Figure 1). Of those, 49 were excluded from the analysis population, with the most common exclusion criterion being absence of a final diagnosis of radiologically confirmed CAP. The median age of study participants was 73 years (interquartile range 60–82), and 67.4% of participants were 65 years or older (Table 1). Most participants (73.4%) had either immunocompromising conditions (IC) or CMC predisposing them to pneumococcal disease. The most frequent ICs among participants were solid malignancies (20.5%), followed by immunosuppressive therapy (12.6%) and chronic kidney disease /end-stage renal disease (9.1%). Chronic obstructive pulmonary disease (COPD) (27.8%), coronary artery disease (26.1%) and congestive heart failure (18.3%) were the most prevalent CMCs among study participants. Current smoking was frequent among participants (18.8%) and higher compared to the Swedish national average of 7% (13). Approximately half of study participants ≥65 years of age had received influenza vaccine within 12 months of study enrolment (48.3%), whereas only 15.7% of participants had ever received any pneumococcal vaccine, and for only one patient could the pneumococcal vaccine that was administered be specified. More details of the demographics and risk factors of the study population are summarized in Supplementary Tables 1, 2.

**Table 1:** Demographics, risk status, pneumonia characteristics and outcomes of participants with radiologically confirmed all-cause CAP.

**Characteristic**	**18–64 years (*n =* 169)**	**≥65 years (*n =* 349)**	**All patients (≥18 years) (*n =* 518)**
Age 18–49 years	77/169 (45.6%)	0/349	77/518 (14.9%)
Age 50–64 years	92/169 (54.4%)	0/349	92/518 (17.8%)
Age 65–79 years	0/169 (0.0%)	187/349 (53.6%)	187/518 (36.1%)
Age ≥80 years	0/169 (0.0%)	162/349 (46.4%)	162/518 (31.3%)
Male sex	92/169 (54.4%)	190/349 (54.4%)	282/518 (54.4%)
White race	144/169 (85.2%)	342/349 (98.0%)	486/518 (93.8%)
Middle east ethnicity	16/169 (9.5%)	3/349 (0.9%)	19/518 (3.7%)
Other race or ethnicity	9/169 (5.3%)	3/349 (0.9%)	12/518 (2.3%)
Mean BMI (kg/m^2^) (SD)	26.3 (5.9)	25.9 (5.4)	26.0 (5.5)
Immunocompromised^a^	39/169 (23.1%)	141/349 (40.4%)	180/518 (34.7%)
Immunosuppression therapy^b^	20/169 (11.8%)	45/347^f^ (13.0%)	65/516v (12.6%)
Immunodeficiency^c^	0/169 (0.0%)	2/348^f^ (0.6%)	2/517^f^ (0.4%)
HIV / AIDS	1/169 (0.6%)	0/348^f^ (0.0%)	1/517^f^ (0.2%)
Cancer/malignancy, solid tumor	17/169 (10.1%)	89/347^f^ (25.6%)	106/516^f^ (20.5%)
Cancer/malignancy, hematologic	4/169 (2.4%)	15/348^f^ (4.3%)	19/517^f^ (3.7%)
Organ transplantation	5/169 (3.0%)	0/348^f^ (0.0%)	5/517^f^ (1.0%)
Chronic kidney disease/end-stage renal disease	6/169 (3.6%)	41/348^f^ (11.8%)	47/517^f^ (9.1%)
Chronic medical conditions^a^	44/169 (26.0%)	156/349 (44.7%)	200/518 (38.6%)
COPD	23/167^f^ (13.8%)	120/347^f^ (34.6%)	143/514^f^ (27.8%)
Asthma	19/169 (11.2%)	28/349 (8.0%)	47/518 (9.1%)
Congestive heart failure	9/169 (5.3%)	86/349 (24.6%)	95/518 (18.3%)
Coronary artery disease	14/169 (8.3%)	121/349 (34.7%)	135/518 (26.1%)
Diabetes mellitus	20/169 (11.8%)	67/349 (19.2%)	87/518 (16.8%)
Liver disease	6/169 (3.6%)	4/349 (1.1%)	10/518 (1.9%)
Autoimmune disorders	13/169 (7.7%)	19/348^f^ (5.5%)	32/517^f^ (6.2%)
Low-risk^a^	86/169 (50.9%)	52/349 (14.9%)	138/518 (26.6%)
Current smoker	46/169 (27.2%)	51/347^f^ (14.7%)	97/516^f^ (18.8%)
Previous smoker	50/169 (29.6%)	184/347^f^ (53.0%)	234/516^f^ (45.3%)
Prior influenza vaccine^d^	13/165^f^ (7.9%)	159/329^f^ (48.3%)	172/494^f^ (34.8%)
Prior pneumococcal vaccine^e^	5/165^f^ (3.0%)	51/324^f^ (15.7%)	56/489^f^ (11.5%)
Nursing home residency prior to admission	0/169 (0.0%)	13/349 (3.7%)	13/518 (2.5%)
PSI Grade IV–V	30/169 (17.8%)	232/349 (66.5%)	262/518 (50.6%)
CRB-65 score 0–1 points	164/169 (97.0%)	257/349 (73.6%)	422/518 (81.5%)
CRB-65 score 2 points	5/169 (3.0%)	77/349 (22.1%)	82/518 (15.8%)
CRB-65 score 3–4 points	0/169 (0.0%)	14/349 (4.0%)	14/518 (2.7%)
Admission to ICU	4/169 (2.4%)	10/349 (2.9%)	14/518 (2.7%)
Duration of hospital stay^a^ (median, IQR)	5 (IQR 3, 6)	7 (IQR 4,10)	6 (IQR 4, 9)
In-hospital CFR	1/169 (0.6%)	18/349 (5.2%)	19/518 (3.7%)
30-day CFR	2/169 (1.2%)	19/349 (5.4%)	21/518 (4.1%)
90-day CFR	3/169 (1.8%)	40/349 (11.5%)	43/518 (8.3%)

a Risk level: Immunocompromised − presence of ≥ 1 immunocompromising condition; underlying chronic medical condition − presence of ≥ 1 chronic medical condition and not immunocompromising condition; low-risk − absence of any immunocompromising or chronic medical conditions.

b Immunosuppression; “prednisolone dos ≥10 mg/day (or equivalent), biological/immunomodulatory or chemotherapy drugs.

c Primary immunodeficiency disorders i.e., IgG-subclass deficiency.

d Prior influenza vaccine – vaccination with influenza vaccine in the year prior to enrollment.

e Prior pneumococcal vaccine – any vaccination with the 23-valent pneumococcal polysaccharide vaccine or the 13-valent pneumococcal conjugate vaccine.

f The denominator is different due to missing information.

CAP, community-acquired pneumonia; COPD, Chronic obstructive pulmonary disease; BMI, body mass index; HIV, human immunodeficiency virus; AIDS, acquired immune deficiency syndrome; PSI Grade, pneumonia severity index grade.

CRB-65 score–score for pneumonia severity: confusion, respiratory rate ≥ 30, systolic BP < 90 mmHg or diastolic BP ≤ 60 mmHg, age ≥ 65; ICU, intensive care unit.

### Disease severity, hospital utilization, and outcomes

Among study participants aged ≥65 years, 66.5% had severe or very severe pneumonia (Pneumonia severity index (PSI) grade IV or V) (14), compared to only 17.8% of younger pneumonia patients (Table 1). Older adults had also longer median duration of hospitalization (7 days in participants ≥ 65 years vs. 5 days in 18–64 years), a higher case fatality ratio (CFR) at 30 days post-admission (5.4 vs. 1.2%) and a higher 90 day-CFR (11.5 vs. 1.8%).

### *Streptococcus pneumoniae* detection rates

**Figure 2:**
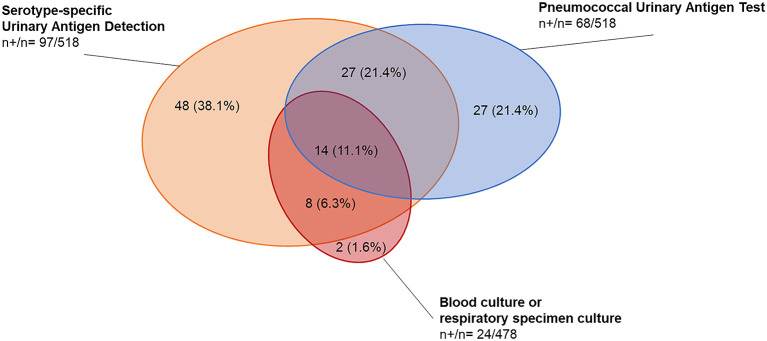
Detection of *S. pneumoniae* in study participants with radiologically confirmed CAP by microbiological test methods. The figure shows the distribution of detected *S. pneumoniae* among 518 study participants with radiologically confirmed CAP and PUAT and UAD results available. n+, number of positive tests; n, total number of tests performed. Percentages indicate the proportion of positives test results per total number of tests among pneumococcal pneumonia detected by diagnostic method (*n* = 126). Culture specimen types included blood (*n* = 22), sputum (*n* = 1) and bronchoalveolar lavage (*n* = 1).

All study participants were tested for Spn by PUAT and UAD and in 91.9% of patients blood cultures were performed. Sputum was collected from 28 (5.4%) patients, pleural fluid samples were obtained from 16 (3.3%) patients and bronchoalveolar lavage, tracheal aspirate or bronchial sterile brush were obtained from 12 (2.3%) patients. Overall, Spn was detected by culture, PUAT, or UAD in 24.3% of CAP cases (Figure 1, Table 2, Supplementary Table 4). Pneumococcal CAP etiology was slightly more frequent in younger participants aged 18–64 years compared to participants aged ≥ 65 years (27.2 vs. 22.9%). Differences were observed in detection rates between the diagnostic tests with overall higher positivity rates for antigen-based assays compared to bacterial cultures (Figure 2). Among the 126 participants with pneumococcal CAP diagnosed by any method, in 19.4% (24 cases) the diagnosis was made by culture-based methods, while urine antigen tests were positive in 98.4% of cases (*n* = 124). Only two cases were identified by culture only. In total, the UAD detected in 97 (77.0%) of pneumococcal CAP and PUAT was positive in 68 (54.0%) of cases. In 48 (38.1%) cases and 27 (21.4%) cases UAD and PUAT were the only positive diagnostic test.

**Table 2:** Pneumococcal etiology, distribution of pneumococcal serotypes and pneumococcal conjugate vaccine coverage among study participants with radiologically confirmed CAP.

	**18–64 years** **(*n* = 169)^a^**		**≥65 years** **(*n* = 349)^a^**		**All patients** **(≥18 years)** **(*n = * 518)^a^**	
	***n***	**%**	***n***	**%**	***n***	**%**
*S. pneumoniae* detected by any method^b^	46	27.2%	80	22.9%	126	24.3%
*S. pneumoniae* w/ serotype information^c^	39	23.1%	60	17.2%	99	19.1%
PCV13 serotypes^d^	21	12.4%	35	10.0%	56	10.8%
3	9	5.3%	17	4.9%	26	5.0%
19A	6	3.6%	4	1.1%	10	1.9%
5	3	1.8%	5	1.4%	8	1.5%
4	0	0.0%	3	0.9%	3	0.6%
6A/6C	0	0.0%	3	0.9%	3	0.6%
14	0	0.0%	2	0.6%	2	0.4%
18C	1	0.6%	1	0.3%	2	0.4%
7F	2	1.2%	0	0.0%	2	0.4%
19F	0	0.0%	1	0.3%	1	0.2%
23F	1	0.6%	0	0.0%	1	0.2%
PCV15 serotypes^d^	23	13.6%	42	12.0%	65	12.5%
PCV15 non-PCV13 serotypes	2	1.2%	7	2.0%	9	1.7%
PCV20 serotypes^d^	35	20.7%	53	15.2%	88	17.0%
PCV20 non-PCV13 serotypes^d^	15	8.9%	18	5.2%	33	6.4%
8	7	4.1%	3	0.9%	10	1.9%
11A	4	2.4%	6	1.7%	10	1.9%
22F	0	0.0%	6	1.7%	6	1.2%
33F	2	1.2%	1	0.3%	3	0.6%
15B/15C	2	1.2%	0	0.0%	2	0.4%
10A	0	0.0%	1	0.3%	1	0.2%
12F	0	0.0%	1	0.3%	1	0.2%
Non-PCV20 serotypes (any method)	5	12.8%	9	15.0%	14	14.1%
Non-PCV20 serotypes (UAD +/- culture)	4	2.4%	7	2.0%	11	2.1%
9N	3	1.8%	4	1.1%	7	1.4%
17F	1	0.6%	3	0.9%	4	0.8%
Non-PCV20 serotypes^e^ (culture only)	1	0.6%	2	0.6%	3	0.6%
Unknown serotype^f^	7	4.1%	20	5.7%	27	5.2%

Four participants were positive for two serotypes. They contribute to counts for multiple serotypes, but only one time to serotypes grouped by PCV formulation. Hence, counts for individual vaccine serotypes may be higher than for grouped vaccine serotypes.

a Number of participants included in the population, with non-missing UAD results. The values in this row are used as the denominators for percentage of participants.

b detection methods include: UAD1/UAD2 (serotypes 4, 6A, 6B, 9V, 14, 18C, 19F, 23F, 1, 5, 7F, 3, 6B, 19A, 22F, 33F, 8, 10A, 11A, 12F, 15B, 2, 9N, 17F, 20 plus cross-reactive serotypes 6C and 15C), PUAT, blood culture or respiratory specimen culture.

c detection methods include: UAD1/UAD2, blood culture or respiratory specimen culture.

d As serotypes 6A and 6C are identified together as 6A/6C in the UAD1 assay and serotypes 15B and 15C are identified together as 15B/15C in the UAD2 assay, the vaccine related serotypes 6C and 15C are included serotype counts grouped by vaccine formulation as appropriate.

e detected by blood culture or respiratory specimen culture only, serotypes not included in UAD1/UAD2 assay.

f pneumococci detected by PUAT only.

CAP, community-acquired pneumonia; PCV, pneumococcal conjugate vaccine; UAD, Pfizer serotype-specific urinary antigen detection test; PUAT, pneumococcal urinary antigen test (BinaxNow *S. pneumoniae*).

Self-reported antibiotic usage within 14 days of study enrolment was recorded in 105 enrolled CAP patients (20.3%). Penicillin V (*n* = 52) was the most used antibiotic, followed by doxycycline (*n* = 29) and amoxicillin (*n* = 19). Culture detection rates for Spn were lower among patients pre-exposed to antibiotics (14.3%) compared to patients without prior antibiotic administration (26.9%). Prior administration of antibiotics was associated with a 46.9% lower detection rate (risk ratio 0.53, 95% confidence interval 0.32–0.87, *p* = 0.007 chi-square test).

### Pneumococcal serotype distribution

In the majority of Spn CAP cases (78.6%), specific serotypes were identified by the UAD test or culture and serotyping. The remaining cases of Spn CAP were diagnosed only by PUAT and the serotype was not established (Table 2). The most frequent serotypes identified were serotype 3 followed by 8, 11A, 19A and 5 (Table 2). Serotypes included in PCV13, PCV15 and PCV20 were detected in 10.8%, 12.5% and 17.0% of adults with CAP, respectively (Table 3). The percentage of all-cause CAP due to serotypes in licensed PCVs is shown in Table 3. Among adults aged 18–64 years, serotypes included in PCV20 contributed 25.0% and 25.6% of all-cause CAP in individuals with CMC or IC, respectively. Moreover, in adults aged ≥65 years, PCV20 serotypes accounted for 15.2% of all-cause CAP.

**Table 3:** Proportion of radiologically confirmed CAP due to pneumococcal serotypes included into PCV13, PCV15, and PCV20.

**Stratum**		**All-cause CAP**			
		**N** ^a^	**PCV13** ^a^	**PCV15** ^a^	**PCV20** ^a^
18–64 years	Any risk	169	21 (12.4%)	23 (13.6%)	35 (20.7%)
	Immunocompromising conditions^b^	39	9 (23.1%)	9 (23.1%)	10 (25.6%)
	Chronic medical conditions^c^	44	3 (6.8%)	4 (9.1%)	11 (25.0%)
	Low-risk^d^	86	9 (10.5%)	10 (11.6%)	14 (16.3%)
≥65 years	Any risk	349	35 (10.0%)	42 (12.0%)	53 (15.2%)
	Immunocompromising conditions^b^	141	16 (11.3%)	18 (12.8%)	24 (17.0%)
	Chronic medical conditions^c^	156	14 (9.0%)	18 (11.5%)	23 (14.7%)
	Low-risk^d^	52	5 (9.6%)	6 (11.5%)	6 (11.5%)
All patients (≥18 years)	Any risk	518	56 (10.8%)	65 (12.5%)	88 (17.0%)
	Immunocompromising conditions^b^	180	25 (13.9%)	27 (15.0%)	34 (18.9%)
	Chronic medical conditions^c^	200	17 (8.5%)	22 (11.0%)	34 (17.0%)
	Low-risk^d^	138	14 (10.1%)	16 (11.6%)	20 (14.5%)

a As serotypes 6A and 6C are identified together as 6A/6C in the UAD1 assay and serotypes 15B and 15C are identified together as 15B/15C in the UAD2 assay, the vaccine related serotypes 6C and 15C are included serotype counts grouped by vaccine formulation as appropriate.

CAP, community-acquired pneumonia; PCV, pneumococcal conjugate vaccine.

b Chronic kidney disease or end-stage renal disease, organ transplantation, immunodeficiency, hematologic or solid tumor malignancy, acquired immunodeficiency syndrome, human immunodeficiency virus, or treatment with immunosuppressive drug therapy including systemic corticosteroids.

c Chronic obstructive pulmonary disease, asthma, congestive heart failure, coronary artery disease, liver disease, diabetes mellitus, and autoimmune disorders without a concurrent immunocompromising condition.

d All other subjects.

## Discussion

The ECAPS study provides the first data from Northern Europe on the distribution of PCV serotypes in hospitalized adults with CAP after introduction of PCVs into the childhood immunization program. Spn remained an important cause of CAP in adults and was detected among a quarter of cases. Moreover, despite robust childhood PCV10 and PCV13 national immunization programs, PCV13, PCV15 and PCV20 serotypes contributed to a relatively high percentage of CAP cases with 11, 13 and 17 % of the cases, respectively. The most frequently identified serotypes in pneumococcal CAP were 3, 8, 11A, and 19A. Current or former smoking was highly prevalent among study participants, and only 15 and 49% of patients with CAP were vaccinated with pneumococcal or influenza vaccines, respectively.

The strengths of our study are the prospective nature, a well-defined study population, a high proportion of microbiologic testing for Spn and complete information on follow up. Limitations also exist. Approximately half of eligible patients could not be enrolled which may have introduced some selection bias toward a healthier and less diverse population due to language barriers and difficulty to obtain consent from severely ill patients. A possible consequence enrolling less severe cases could be lower test positivity of antigen tests, although due to the lack of gold standard diagnostic tests, the performance of UAD and PUAT for non-bacteremic CAP is difficult to assess. Vaccine-probe studies suggest that the UAD likely underestimates the true proportion of PCV-preventable CAP (15). Also, with lower disease severity antigenuria may have subsided already at the time urine collection, leading to false-negative results. Lastly, the UAD test is likely more sensitive than PUAT but only includes 24 of the more than 100 known Spn serotypes in the former assay. Overall pneumococcal detection might have been considerably higher if the UAD tests had covered all known serotypes. As PUAT as a pan-pneumococcal antigen test is less sensitive than the UAD for the detection of Spn, the proportion of pneumococcal CAP due to non-PCV serotypes is likely underestimated.

Spn was identified in 24% of CAP cases in our study, a proportion slightly lower than other European studies, such as the CAPA study from Spain (29%) (10), or a prospective pneumonia surveillance study from the UK (37%) (4). In the two latter studies, however, lower respiratory tract sampling was performed much more frequently than in our study. When using the same diagnostic work-up as in Sweden, Spn was identified in 10–13% of US adults hospitalized with CAP (16–18) confirming previous observations of greater identification of Spn among CAP patients in Europe compared to the US (19). Spn was slightly more frequent in younger participants compared to patients aged ≥65 years. A possible explanation could be a lower threshold for hospital admission or more severe clinical presentation of viral pneumonia in older compared to younger adults.

In southern Sweden, where the ECAPS study was performed, PCV7 was replaced by PCV10 in 2010 and in 2014 the program switched to PCV13. In May 2018 a further switch was done to PCV10 albeit there was a transition period for some additional months (20). Despite PCV13 use in children, PCV13 serotypes were still associated with 11% of hospitalized CAP among adults, with contributions from 10 serotypes included in PCV13 and serotypes 3 and 19A combined accounting for 7% of all CAP cases. Other studies using the serotype specific UAD assay also reported that PCV13 serotypes continued to account for 8–13% of CAP hospitalizations in adults in European (4, 10, 21). This illustrates the limits of protecting adults through the indirect effects afforded by childhood PCV programs vs. directly vaccinating adult populations with PCVs which are effective against non-bacteremic pneumonia (22). In the ECAPS cohort, 6% of CAP patients were positive for the additional PCV20 serotypes 8, 11A, 22F, 33F 15B/C, 10A, and 12F. This is within the range of adult CAP attributed to PCV20 unique serotypes reported from Germany (5%) (21), Spain (11%) (10), and the UK (12%) (4), but higher than in the US (2%) (16).

As pneumococcal serotype 3 was the most frequently identified serotype in adult CAP in southern Sweden, it is critical that vaccines prevent disease due to this serotype. Several lines of evidence support PCV13 effectiveness against serotype 3 in directly vaccinated older adults (23, 24). Additionally, effectiveness against serotype 3 IPD in European children below age 5 years was confirmed by the EU SpIDnet multicenter study, although effectiveness was lower compared to other PCV13 serotypes (25). Because PCV13 may have relatively limited impact on serotype 3 carriage (26, 27), and indirect protection of older adults from pediatric immunization may be less than for other serotypes, direct vaccination of adults assumes greater importance for preventing serotype 3 disease.

The public health impact of pneumococcal vaccination not only depends on the relative frequency of serotypes in disease but also on their contributions to severe disease outcomes and their propensity to cause disease in vulnerable populations. Although not analyzed in our study due to limited sample size, it has been shown that of the most prevalent serotypes in Swedish CAP patients, serotype 8 has been shown to be highly invasive primarily in adults (28–30), serotype 3 and 19 A are associated with higher severity and complication rate in CAP (10, 31, 32), and serotypes 3, 11A, and 19A are associated with higher case-fatality ratios (32–34). Also, individuals with CMC or immunocompromising conditions have an increased relative risk for IPD due to non-PCV13 serotypes and serotypes 2, 20, 9N, 10A, 11A, 8, 12F, 15B, 22F, 33F in particular, most of which are included in the PCV20 formulation (35).

Our data illustrate a persistent burden of adult pneumonia due to PCV10 and PCV13 serotypes despite the use of PCVs in Sweden's national pediatric immunization program. PCV15 and PCV20 will expand coverage against serotypes commonly identified in pneumococcal CAP. If adult PCV vaccination is found to be an efficient use of resources, it will be critical to improve uptake in Sweden, which was low in our cohort as well on a national level as reported elsewhere (3, 35). A significant portion of the participants were smokers. This fact emphasizes the importance of smoking cessation programs, together with improved implementation of influenza and pneumococcal vaccines in disease preventing work.

## Author's note

This work has been previously presented at the 12th International Symposium on Pneumococci and Pneumococcal Diseases (ISPPD), in Toronto, Canada on 19–23 June 2022.

## Data availability statement

The original contributions presented in the study are included in the article/Supplementary material, further inquiries can be directed to the corresponding authors.

## Ethics statement

This study was approved by the Lund Regional Ethics Committee (Nos. 2016/220 and 2016/340). The patients/participants provided their written informed consent to participate in this study.

## Author contributions

KH, ER, RB, KR, and JA designed and coordinated the study. KH and ER acquired the data. QJ analyzed the data by contribution of GT. CT drafted the manuscript. All authors interpreted the data and edited, critically revised, and approved the final manuscript.
