# Supplementary material for: Radiographically confirmed community-acquired pneumonia in hospitalized adults due to pneumococcal vaccine serotypes in Sweden, 2016–2018—The ECAPS study
Source: Front Public Health. 2023 Feb 17;11:1086648. doi: 10.3389/fpubh.2023.1086648 (PMC9981934; doi:10.3389/fpubh.2023.1086648)
Supplement: Supplementary file 1 [file Data_Sheet_1.pdf]

Supplemental Methods and Materials

Pneumococcal etiology and serotype distribution among adults hospitalized with radiographically confirmed Community-Acquired Pneumonia in Sweden, 2016-2018 – the ECAPS study

Karin Hansen[1,2†], Elisabeth Rünow[1,2†], Gustav Torisson[1], Christian Theilacker[3], Andreas Palmborg[4], Kaijie Pan[3], Qin Jiang[3], Jo Southern[3], Rohini Beavon[3], Bradford D. Gessner[3], Kristian Riesbeck[2#], Jonas Ahl[1,2#]

[1]Section of Infectious Diseases and [2]Clinical Microbiology, Dept. Translational Medicine, Faculty of Medicine, Lund University, Malmö, Sweden
[3]Vaccines Medical Development, Scientific and Clinical Affairs, Pfizer, Collegeville, PA
[4]Pfizer Vaccines, Medical Development, Scientific and Clinical Affairs, Stockholm, Sweden
Corresponding authors: Jonas Ahl and Kristian Riesbeck

[†] These authors contributed equally to this work and share first authorship
[#] These authors contributed equally to this work and share last authorship

Primary objective

To estimate the proportion of *S. pneumoniae* serotypes included in PCV13 among adults ≥18 years of age presenting with radiographically-confirmed CAP.

Secondary objectives

1. To determine the full distribution of all *S. pneumoniae* serotypes among adults with CAP.
2. To estimate the incidence rate of CAP and of *S. pneumoniae* positive radiologically-confirmed CAP (SP+ CAP).
3. To determine the differences in detection of *S. pneumoniae* by culture, BinaxNOW® and the UAD assay.
4. To calculate the proportion of subjects with CAP and with SP+CAP who present with underlying at-risk and high-risk medical conditions
5. To estimate antibiotic resistance rates among isolates of *S. pneumonia* in adult CAP in Malmö, Sweden.

Screening criteria

All adult patients admitted between September 2016 16th to September 16th 2018 to the emergency department of Skåne University Hospital were pre-screened by study staff for an ordered radiologic imaging study. Those with a radiologic imaging study of the chest ordered, were assessed for meeting the study inclusion and exclusion criteria (see below).

Inclusion criteria

1. Age ≥ 18 years.
2. Present to a study healthcare facility where the treating physician clinically suspects CAP with the presence of two or more of the following signs or symptoms:
    - Fever (oral temperature > 38°C or tympanic temperature > 38.5°C) within 24 hours before enrollment
    - Hypothermia (<35.5°C measured by a healthcare provider) within 24 hours of enrollment
    - Chills or rigors
    - Pleuritic chest pain
    - Cough
    - Sputum production
    - Dyspnea (shortness of breath)
    - Tachypnea (respiratory rate > 20/ min)
    - Malaise
    - Abnormal auscultatory findings suggestive of pneumonia (rales or evidence of pulmonary consolidation (dullness on percussion, bronchial breath sounds, or egophony).
3. Has a radiographic finding that is consistent with pneumonia. (e.g. pleural effusion, increased pulmonary density due to infection, the presence of alveolar infiltrates (multilobar, lobar or segmental) containing air bronchograms, etc.). Chest radiographs will be used to meet the inclusion criterion. Chest computed tomography (CT) scans or other relevant radiological tests performed as part of local standard of care may be used instead of a chest x-ray to assess subject eligibility.

4. Able and willing to provide urine sample(s).
5. Evidence of a personally signed and dated informed consent document indicating that the subject (or a legal representative) has been informed of all pertinent aspects of the study.
6. Resident of catchment area of Malmö, Vellinge and Svedala.

Exclusion criteria

1. Subjects hospitalized for 48 hours or more or transferred to a study healthcare facility after already being hospitalized for 48 hours or more at any other in-patient facility (such as a community hospital).
2. Hospital acquired pneumonia (e.g. develops signs and symptoms of pneumonia after being hospitalized for 48 hours or more) during the previous 30 days.
3. Subjects who are investigational site staff members directly involved in the conduct of the trial and their family members; site staff members otherwise supervised by the Investigator.
4. Previous enrollment in this study within the previous 30 days.
5. Other severe acute or chronic medical or psychiatric condition or laboratory abnormality that may increase the risk associated with study participation or may interfere with the interpretation of study results and, in the judgment of the Investigator, would make the subject inappropriate for entry into this study.

Microbiologic specimen collection

Blood cultures were collected at admission to the ED before administration of intravenous antibiotics. If a blood culture was not obtained as part of the local standard of care this procedure was conducted per protocol within 24 hours of study enrollment. Urine samples were collected per-protocol from all participants preferably withing the first 24 h of enrolment. In addition, culture results from blood, pleural puncture and lower respiratory tract specimens collected as standard of care were also recorded.

Microbiological diagnosis and testing

Blood culture, respiratory tract specimens and pleural fluid was cultured in the local laboratory according to standard methods. Serotyping was performed using a multiplex polymerase chain reaction (PCR) comprising 6 sequential reactions in combination with latex agglutination and the Quellung reaction as described [1]. Isolates that were negative twice in PCRs were serotyped with the ImmuLex Pneumotest Kit (SSI Diagnostica, Copenhagen, DK) and Neufeldt antisera. Isolates that were negative for *cpsA* in PCR twice or could not be determined through latex agglutination and the Quellung reaction, were considered non-typeable (NT).

Urine testing

Urine specimens were tested by BinaxNOW *S. pneumoniae*® (a commercially available testing kit for the identification of *S pneumoniae;* Abbott Diagnostics, Scarborough, ME, USA), UAD1, and UAD2 at Pfizer's Vaccines Research and Development Laboratory (Pearl River, NY, USA) [2, 3]. The UAD assay is a limit assay that uses Luminex technology, with positivity cutoff limits (based on antigen concentrations read off a standard curve), established for each serotype using 400 control urine specimens collected from otherwise healthy adults without CAP. Using

nonparametric tolerance intervals, the assay is set to achieve at least 97% specificity for each serotype.

Study procedures and assessments

Participants were classified as immunocompromised if they had chronic kidney disease or end-stage renal disease, organ transplantation, immunodeficiency, hematologic or solid tumor malignancy, acquired immunodeficiency syndrome, human immunodeficiency virus, or were treated with immunosuppressive drug therapy including systemic corticosteroids. Chronic underlying medical conditions predisposing to pneumococcal disease were defined as chronic obstructive pulmonary disease, asthma, congestive heart failure, coronary artery disease, liver disease, diabetes mellitus, and autoimmune disorders without a concurrent immunocompromising condition. All other subjects were classified as low-risk. These categories were based on current ACIP recommendations for pneumococcal vaccination [4]. A final assessment was made after discharge by study investigators based on all relevant data and assessments available.

Pneumonia severity was assessed by using Pneumonia severity index (PSI), a prediction score including age, sex, medical history and findings from both physical and laboratory examination. A score between 0-405 is grouped in to 5 classes, I-V, were risk class V being the highest risk of mortality [5].

Antibiotic therapy within the last 14 days was documented as reported by the patient.

Data analysis

Patients without a diagnosis of pneumonia but an alternative diagnosis explaining the symptoms and radiological findings at inclusion were excluded from the analysis. A participant may have had up to 2 different serotypes identified from UAD-1 and UAD-2, respectively, totaling a maximum of 4 different serotypes from UADs per participant. Similarly, it was also possible that multiple serotypes per participant were identified by culture. In the case of multiple serotype detection per participant, each serotype contributed to counts reported for individual serotypes, but for serotypes grouped by vaccine formulation, multiple serotypes belonging to the same vaccine formulation contributed only once. Thus, the counts for individual vaccine serotypes may exceed the counts for serotypes grouped by vaccine.

Supplemental References

1. Udden F, Filipe M, Reimer A, Paul M, Matuschek E, Thegerstrom J, Hammerschmidt S, Pelkonen T, Riesbeck K. Aerobic bacteria associated with chronic suppurative otitis media in Angola. Infectious diseases of poverty **2018**; 7(1): 42.
2. Pride MW, Huijts SM, Wu K, Souza V, Passador S, Tinder C, Song E, Elfassy A, McNeil L, Menton R, French R, Callahan J, Webber C, Gruber WC, Bonten MJ, Jansen KU. Validation of an immunodiagnostic assay for detection of 13 Streptococcus pneumoniae serotype-specific polysaccharides in human urine. Clin Vaccine Immunol **2012**; 19(8): 1131-41.
3. Kalina WV, Souza V, Wu K, Giardina P, McKeen A, Jiang Q, Tan C, French R, Ren Y, Belanger K, McElhiney S, Unnithan M, Cheng H, Mininni T, Giordano-Schmidt D, Gessner BD, Jansen KU, Pride MW. Qualification and Clinical Validation of an Immunodiagnostic Assay for Detecting 11 Additional Streptococcus pneumoniae Serotype-specific Polysaccharides in Human Urine. Clin Infect Dis **2020**; 71(9): e430-e8.
4. Kobayashi M, Farrar JL, Gierke R, Britton A, Childs L, Leidner AJ, Campos-Outcalt D, Morgan RL, Long SS, Talbot HK, Poehling KA, Pilishvili T. Use of 15-Valent Pneumococcal Conjugate Vaccine and 20-Valent Pneumococcal Conjugate Vaccine Among U.S. Adults: Updated Recommendations of the Advisory Committee on

Immunization Practices - United States, 2022. MMWR Morb Mortal Wkly Rep **2022**; 71(4): 109-17.

5. Fine MJ, Auble TE, Yealy DM, Hanusa BH, Weissfeld LA, Singer DE, Coley CM, Marrie TJ, Kapoor WN. A prediction rule to identify low-risk patients with community-acquired pneumonia. N Engl J Med. **1997**;336(4):243-50

Supplemental Results

Supplemental Table 1 Demographics by Age Groups and *S. pneumoniae* Positivity Status – Radiologically-Confirmed CAP Population

	18 - < 65 Years			≥65 Years			Total		
	Sp+ (N=46)	Sp- (N=123)	All CAP (N=169)	Sp+ (N=80)	Sp- (N=269)	All CAP (N=349)	Sp+ (N=126)	Sp- (N=392)	All CAP (N=518)
Age at consent (years)									
n	46	123	169	80	269	349	126	392	518
Mean (SD)	50.3 (11.96)	47.6 (13.19)	48.3 (12.89)	78.5 (8.08)	79.2 (7.97)	79.1 (7.99)	68.2 (16.72)	69.3 (17.73)	69.0 (17.48)
Median	56.5	52.0	52.0	78.0	79.0	79.0	70.0	73.0	73.0
Min, Max	22, 64	18, 64	18, 64	65, 96	65, 99	65, 99	22, 96	18, 99	18, 99
Age category									
18 - < 65 years	46 (100.0%)	123 (100.0%)	169 (100.0%)	0 (0.0%)	0 (0.0%)	0 (0.0%)	46 (36.5%)	123 (31.4%)	169 (32.6%)
18 - < 50 years	19 (41.3%)	58 (47.2%)	77 (45.6%)	0 (0.0%)	0 (0.0%)	0 (0.0%)	19 (15.1%)	58 (14.8%)	77 (14.9%)
50 - < 65 years	27 (58.7%)	65 (52.8%)	92 (54.4%)	0 (0.0%)	0 (0.0%)	0 (0.0%)	27 (21.4%)	65 (16.6%)	92 (17.8%)
≥ 65 years	0 (0.0%)	0 (0.0%)	0 (0.0%)	80 (100.0%)	269 (100.0%)	349 (100.0%)	80 (63.5%)	269 (68.6%)	349 (67.4%)
65 - < 80 years	0 (0.0%)	0 (0.0%)	0 (0.0%)	45 (56.3%)	142 (52.8%)	187 (53.6%)	45 (35.7%)	142 (36.2%)	187 (36.1%)
≥ 80 years	0 (0.0%)	0 (0.0%)	0 (0.0%)	35 (43.8%)	127 (47.2%)	162 (46.4%)	35 (27.8%)	127 (32.4%)	162 (31.3%)
Sex									
Male	26 (56.5%)	66 (53.7%)	92 (54.4%)	46 (57.5%)	144 (53.5%)	190 (54.4%)	72 (57.1%)	210 (53.6%)	282 (54.4%)
Female	20 (43.5%)	57 (46.3%)	77 (45.6%)	34 (42.5%)	125 (46.5%)	159 (45.6%)	54 (42.9%)	182 (46.4%)	236 (45.6%)
Race									
White	40 (87.0%)	104 (84.6%)	144 (85.2%)	78 (97.5%)	264 (98.1%)	342 (98.0%)	118 (93.7%)	368 (93.9%)	486 (93.8%)
Middle east	3 (6.5%)	13 (10.6%)	16 (9.5%)	1 (1.3%)	2 (0.7%)	3 (0.9%)	4 (3.2%)	15 (3.8%)	19 (3.7%)
Hispanic	2 (4.3%)	3 (2.4%)	5 (3.0%)	0 (0.0%)	1 (0.4%)	1 (0.3%)	2 (1.6%)	4 (1.0%)	6 (1.2%)
Black	0 (0.0%)	2 (1.6%)	2 (1.2%)	0 (0.0%)	2 (0.7%)	2 (0.6%)	0 (0.0%)	4 (1.0%)	4 (0.8%)
Asia	1 (2.2%)	1 (0.8%)	2 (1.2%)	0 (0.0%)	0 (0.0%)	0 (0.0%)	1 (0.8%)	1 (0.3%)	2 (0.4%)
Missing	0 (0.0%)	0 (0.0%)	0 (0.0%)	1 (1.3%)	0 (0.0%)	1 (0.3%)	1 (0.8%)	0 (0.0%)	1 (0.2%)

Supplemental Table 1 Demographics by Age Groups and *S. pneumoniae* Positivity Status – Radiologically-Confirmed CAP Population

	**18 - < 65 Years**			**≥65 Years**			**Total**		
	**Sp+ (N=46)**	**Sp- (N=123)**	**All CAP (N=169)**	**Sp+ (N=80)**	**Sp- (N=269)**	**All CAP (N=349)**	**Sp+ (N=126)**	**Sp- (N=392)**	**All CAP (N=518)**
Height (cm)									
n	46	123	169	80	269	349	126	392	518
Mean (SD)	171.3 (7.72)	172.5 (8.39)	172.2 (8.20)	170.1 (9.67)	169.1 (9.73)	169.3 (9.71)	170.5 (9.00)	170.2 (9.45)	170.3 (9.33)
Median	172.5	172.0	172.0	171.5	170.0	170.0	172.0	170.0	170.0
Min, Max	150, 188	150, 190	150, 190	138, 191	143, 196	138, 196	138, 191	143, 196	138, 196
Weight (kg)									
n	46	123	169	80	269	349	126	392	518
Mean (SD)	76.4 (17.55)	78.4 (18.33)	77.8 (18.09)	70.5 (15.18)	75.9 (18.35)	74.7 (17.80)	72.6 (16.27)	76.7 (18.35)	75.7 (17.94)
Median	72.5	80.0	78.0	71.0	74.0	73.0	71.5	75.0	75.0
Min, Max	43, 111	43, 130	43, 130	38, 115	35, 140	35, 140	38, 115	35, 140	35, 140
BMI (kg/m^2)									
n	46	123	169	80	269	349	126	392	518
Mean (SD)	26.1 (5.81)	26.4 (5.90)	26.3 (5.86)	24.3 (4.88)	26.4 (5.40)	25.9 (5.36)	24.9 (5.29)	26.4 (5.56)	26.0 (5.52)
Median	25.0	26.0	26.0	24.0	26.0	25.0	24.0	26.0	25.0
Min, Max	15, 46	14, 43	14, 46	16, 39	13, 45	13, 45	15, 46	13, 45	13, 46
Swedish citizen [n (%)]	46 (100.0%)	123 (100.0%)	169 (100.0%)	80 (100.0%)	269 (100.0%)	349 (100.0%)	126 (100.0%)	392 (100.0%)	518 (100.0%)

Abbreviations: Sp+ - *S. pneumoniae* positive, SD - standard deviation.

Supplemental Table 2 Medical History by Age Groups and *S. pneumoniae* Positivity Status – Radiologically-Confirmed CAP Population

	**18-<65 Years**			**≥ 65 Years**			**≥18 Years**		
	**Sp+ (N=46)**	**Sp- (N=123)**	**All CAP (N=169)**	**Sp+ (N=80)**	**Sp- (N=269)**	**All CAP (N=349)**	**Sp+ (N=126)**	**Sp- (N=392)**	**All CAP (N=518)**
Risk level[1]									
High risk	12/46 (26.1%)	27/123 (22.0%)	39/169 (23.1%)	33/80 (41.3%)	108/269 (40.2%)	141/349 (40.4%)	45/126 (35.7%)	135/392 (34.4%)	180/518 (34.8%)
At risk	15/46 (32.6%)	29/123 (23.6%)	44/169 (26.0%)	35/80 (43.8%)	121/269 (45.0%)	156/349 (44.7%)	50/126 (39.7%)	150/392 (38.3%)	200/518 (38.6%)
Low risk[1]	19/46 (41.3%)	67/123 (54.5%)	86/169 (50.9%)	12/80 (15.0%)	40/269 (14.9%)	52/349 (14.9%)	31/126 (24.6%)	107/392 (27.3%)	138/518 (26.6%)
High risk									
Immunosuppression therapy	5/46 (10.9%)	15/123 (12.2%)	20/169 (11.8%)	10/80 (12.5%)	35/267 (13.1%)	45/347 (13.0%)	15/126 (11.9%)	50/390 (12.8%)	65/516 (12.6%)
Chronic kidney disease incl. ESRD	3/46 (6.5%)	3/123 (2.4%)	6/169 (3.6%)	6/80 (7.5%)	35/268 (13.1%)	41/348 (11.8%)	9/126 (7.1%)	38/391 (9.7%)	47/517 (9.1%)
Immunodeficiency	0/46 (0.0%)	0/123 (0.0%)	0/169 (0.0%)	0/80 (0.0%)	2/268 (0.8%)	2/348 (0.6%)	0/126 (0.0%)	2/391 (0.5%)	2/517 (0.4%)
HIV	1/46 (2.2%)	0/123 (0.0%)	1/169 (0.6%)	0/80 (0.0%)	0/268 (0.0%)	0/348 (0.0%)	1/126 (0.8%)	0/391 (0.0%)	1/517 (0.2%)
AIDS	0/46 (0.0%)	0/123 (0.0%)	0/169 (0.0%)	0/80 (0.0%)	0/268 (0.0%)	0/348 (0.0%)	0/126 (0.0%)	0/391 (0.0%)	0/517 (0.0%)
Cancer/malignancy, solid tumor	7/46 (15.2%)	10/123 (8.1%)	17/169 (10.1%)	25/80 (31.3%)	64/267 (24.0%)	89/347 (25.7%)	32/126 (25.4%)	74/390 (19.0%)	106/516 (20.6%)
Cancer/malignancy, hematologic	0/46 (0.0%)	4/123 (3.3%)	4/169 (2.4%)	5/80 (6.3%)	10/268 (3.8%)	15/348 (4.3%)	5/126 (4.0%)	14/391 (3.6%)	19/517 (3.7%)
Organ transplantation	2/46 (4.4%)	3/123 (2.4%)	5/169 (3.0%)	0/80 (0.0%)	0/268 (0.0%)	0/348 (0.0%)	2/126 (1.6%)	3/391 (0.8%)	5/517 (1.0%)
At risk									
COPD	9/46 (19.6%)	14/121 (11.6%)	23/167 (13.8%)	32/79 (40.5%)	88/268 (32.8%)	120/347 (34.6%)	41/125 (32.8%)	102/389 (26.2%)	143/514 (27.9%)

Supplemental Table 2 Medical History by Age Groups and *S. pneumoniae* Positivity Status – Radiologically-Confirmed CAP Population

	18-<65 Years			≥ 65 Years			≥18 Years		
	**Sp+ (N=46)**	**Sp- (N=123)**	**All CAP (N=169)**	**Sp+ (N=80)**	**Sp- (N=269)**	**All CAP (N=349)**	**Sp+ (N=126)**	**Sp- (N=392)**	**All CAP (N=518)**
Asthma	8/46 (17.4%)	11/123 (9.0%)	19/169 (11.2%)	3/80 (3.8%)	25/269 (9.3%)	28/349 (8.0%)	11/126 (8.7%)	36/392 (9.2%)	47/518 (9.1%)
Congestive heart failure	1/46 (2.2%)	8/123 (6.5%)	9/169 (5.3%)	17/80 (21.3%)	69/269 (25.7%)	86/349 (24.6%)	18/126 (14.3%)	77/392 (19.6%)	95/518 (18.3%)
Coronary artery disease	4/46 (8.7%)	10/123 (8.1%)	14/169 (8.3%)	33/80 (41.3%)	88/269 (32.7%)	121/349 (34.7%)	37/126 (29.4%)	98/392 (25.0%)	135/518 (26.1%)
Autoimmune disorders	5/46 (10.9%)	8/123 (6.5%)	13/169 (7.7%)	1/80 (1.3%)	18/268 (6.7%)	19/348 (5.5%)	6/126 (4.8%)	26/391 (6.7%)	32/517 (6.2%)
Diabetes mellitus	5/46 (10.9%)	15/123 (12.2%)	20/169 (11.8%)	13/80 (16.3%)	54/269 (20.1%)	67/349 (19.2%)	18/126 (14.3%)	69/392 (17.6%)	87/518 (16.8%)
Liver disease	2/46 (4.4%)	4/123 (3.3%)	6/169 (3.6%)	2/80 (2.5%)	2/269 (0.7%)	4/349 (1.2%)	4/126 (3.2%)	6/392 (1.5%)	10/518 (1.9%)

N=total number of pathogens identified from the specified test.
n=Total counts with positive specified pathogen.
Abbreviations: Sp+ - *S. pneumoniae* positive, ESRD – end-stage renal disease, COPD – chronic obstructive pulmonary disease, HIV – human immunodeficiency virus, AIDS – acquired immunodeficiency syndrome

[1] Low risk: All subjects not defined as High or At risk.

Supplemental Table 3 *Streptococcus pneumonia* (Sp+) from Diagnostic Method by Age Groups and *S. pneumoniae* Positivity Status – Radiologically-Confirmed CAP Population

SP+ Diagnostic	CXR+CAP Population [n /N (%)]			Sp+ CXR+CAP Subpopulation [% (n/N)]		
	18 - < 65 years	≥ 65 years	All	18 - < 65 years	≥ 65 years	All
SP+ from any diagnostic method [a]	46/169 (27.2%)	80/349 (22.9%)	126/518 (24.3%)	46/46 (100.0%)	80/80 (100.0%)	126/126 (100.0%)
1: UAD alone	17/169 (10.1%)	31/349 (8.9%)	48/518 (9.3%)	17/46 (37.0%)	31/80 (38.8%)	48/126 (38.1%)
2: UAD + Binax	11/169 (6.5%)	16/349 (4.6%)	27/518 (5.2%)	11/46 (23.9%)	16/80 (20.0%)	27/126 (21.4%)
3: Binax alone	7/169 (4.1%)	20/349 (5.7%)	27/518 (5.2%)	7/46 (15.2%)	20/80 (25.0%)	27/126 (21.4%)
4: Binax + Culture	0/169 (0.0%)	0/349 (0.0%)	0/518 (0.0%)	0/46 (0.0%)	0/80 (0.0%)	0/126 (0.0%)
5: UAD + Culture	3/169 (1.8%)	5/349 (1.4%)	8/518 (1.5%)	3/46 (6.5%)	5/80 (6.3%)	8/126 (6.3%)
6: Culture alone	1/169 (0.6%)	1/349 (0.3%)	2/518 (0.4%)	1/46 (2.2%)	1/80 (1.3%)	2/126 (1.6%)
7: UAD + Binax + Culture	7/169 (4.1%)	7/349 (2.0%)	14/518 (2.7%)	7/46 (15.2%)	7/80 (8.8%)	14/126 (11.1%)
SP+ from UAD [b]	38/169 (22.5%)	59/349 (16.9%)	97/518 (18.7%)	38/46 (82.6%)	59/80 (73.8%)	97/126 (77.0%)
SP+ from BinaxNOW [c]	25/169 (14.8%)	43/349 (12.3%)	68/518 (13.1%)	25/46 (54.3%)	43/80 (53.8%)	68/126 (54.0%)
SP+ from Sterile Culture [d]	11/165 (6.7%)	11/311 (3.5%)	22/476 (4.6%)	11/45 (24.4%)	11/74 (14.9%)	22/119 (18.5%)
SP+ from non-Sterile Culture [e]	0/15 (0.0%)	2/32 (6.3%)	2/47 (4.3%)	0/3 (0.0%)	2/3 (66.7%)	2/6 (33.3%)
SP+ from Any Culture [f]	11/166 (6.6%)	13/312 (4.2%)	24/478 (5.0%)	11/45 (24.4%)	13/74 (17.6%)	24/119 (20.2%)
SP+ from any Culture or BinaxNOW [g]	29/169 (17.2%)	49/349 (14.0%)	78/518 (15.1%)	29/46 (63.0%)	49/80 (61.3%)	78/126 (61.9%)

Note: The 3 diagnostic methods are UAD, culture, and BinaxNOW.
a. Item 1-7 are mutually exclusive. N = number of subjects included in the specified population and with non-missing results from at least 1 diagnostic methods.
b. N = number of subjects included in the specified population and with non-missing UAD results. The count for n in this row is the total from item 1,2,5,7.
c. N = number of subjects included in the specified population and with non-missing BinaxNOW results. The count for n in this row is the total from item 2,3,4,7.
d. N = number of subjects included in the specified population and with non-missing sterile culture results.
e. N = number of subjects included in the specified population and with non-missing non-sterile culture results.
f. N = number of subjects included in the specified population and with non-missing culture results.
g. N = number of subjects included in the specified population and with non-missing BinaxNOW and non-missing culture results.
Abbreviations: Sp+ - *S. pneumoniae* positive, CXR+ - radiologically confirmed, UAD – Urinary antigen detection assay

Supplemental Table 4 Case-fatality risk and proportion of radiologically confirmed CAP due to pneumococcal serotypes included into PCV13, PCV15 and PCV20. by risk status

		**Case-fatality risk**			**PCV Formulation**						
**Categories Subcategories**	**N**	**In-hospital n (%)**	**30-day n (%)**	**With 90-day n (%)**	**Sp+ n (%)**	**PCV7 n (%)**	**PCV13-PCV7 n (%)**	**PCV13 n (%)**	**PCV15 n (%)**	**PCV20 n (%)**	**PCV20-PCV13 n (%)**
≥18-64 Years	169	1 (0.6%)	2 (1.2%)	3 (1.8%)	46 (27.2%)	2 (1.2%)	20 (11.8%)	21 (12.4%)	23 (13.6%)	35 (20.7%)	15 (8.9%)
IC	39	0 (0.0%)	0 (0.0%)	0 (0.0%)	12 (30.8%)	1 (2.6%)	9 (23.1%)	9 (23.1%)	9 (23.1%)	10 (25.6%)	1 (2.6%)
CMC	44	0 (0.0%)	0 (0.0%)	1 (2.3%)	15 (34.1%)	0 (0.0%)	3 (6.8%)	3 (6.8%)	4 (9.1%)	11 (25.0%)	9 (20.5%)
Low risk	86	1 (1.2%)	2 (2.3%)	2 (2.3%)	19 (22.1%)	1 (1.2%)	8 (9.3%)	9 (10.5%)	10 (11.6%)	14 (16.3%)	5 (5.8%)
≥65 Years	349	18 (5.2%)	19 (5.4%)	40 (11.5%)	80 (22.9%)	7 (2.0%)	28 (8.0%)	35 (10.0%)	42 (12.0%)	53 (15.2%)	18 (5.2%)
IC	141	9 (6.4%)	9 (6.4%)	20 (14.2%)	33 (23.4%)	2 (1.4%)	14 (9.9%)	16 (11.3%)	18 (12.8%)	24 (17.0%)	8 (5.7%)
CMC	156	7 (4.5%)	7 (4.5%)	15 (9.6%)	35 (22.4%)	4 (2.6%)	10 (6.4%)	14 (9.0%)	18 (11.5%)	23 (14.7%)	9 (5.8%)
Low risk	52	2 (3.8%)	3 (5.8%)	5 (9.6%)	12 (23.1%)	1 (1.9%)	4 (7.7%)	5 (9.6%)	6 (11.5%)	6 (11.5%)	1 (1.9%)
All ≥18 Years	518	19 (3.7%)	21 (4.1%)	43 (8.3%)	126 (24.3%)	9 (1.7%)	48 (9.3%)	56 (10.8%)	65 (12.5%)	88 (17.0%)	33 (6.4%)
IC	180	9 (5.0%)	9 (5.0%)	20 (11.1%)	45 (25.0%)	3 (1.7%)	23 (12.8%)	25 (13.9%)	27 (15.0%)	34 (18.9%)	9 (5.0%)
CMC	200	7 (3.5%)	7 (3.5%)	16 (8.0%)	50 (25.0%)	4 (2.0%)	13 (6.5%)	17 (8.5%)	22 (11.0%)	34 (17.0%)	18 (9.0%)
Low risk	138	3 (2.2%)	5 (3.6%)	7 (5.1%)	31 (22.5%)	2 (1.4%)	12 (8.7%)	14 (10.1%)	16 (11.6%)	20 (14.5%)	6 (4.3%)
Sex	0										
Male	282	16 (5.7%)	18 (6.4%)	30 (10.6%)	72 (25.5%)	1 (0.4%)	33 (11.7%)	34 (12.1%)	37 (13.1%)	51 (18.1%)	18 (6.4%)
Female	236	3 (1.3%)	3 (1.3%)	13 (5.5%)	54 (22.9%)	8 (3.4%)	15 (6.4%)	22 (9.3%)	28 (11.9%)	37 (15.7%)	15 (6.4%)

Note: n = Number of subjects with condition present; N = number of subjects answered 'Yes' or 'No'. For each individual item, 'Unknown' or 'Not Assessed' are not included in the denominator (N).
Abbreviations: CAP – community-acquired pneumonia, IC – immunocompromising conditions, CMC – chronic medical conditions
